# Beyond the genome: epigenetic regulation of immune responses and T cells in brain tumors

**DOI:** 10.3389/fimmu.2025.1690552

**Published:** 2025-11-18

**Authors:** Shuo Sun, Yu Han, Haiying Li, Chengyan Wang, Shu Zhou, Xiaowei Zhang, Shuhong Dai, Yao Peng, Zhuoqun Wang

**Affiliations:** 1 Department of Neurosurgery, Zibo Central Hospital, No. 54, Gong Qing Tuan Xi Road, Zibo, China; 2 Department of Ultrasound, Jilin Cancer Hospital, Changchun, Jilin, China; 3 Department of Anesthesiology, Jilin Cancer Hospital, Changchun, Jilin, China; 4 Department of Orthopedics, Zibo Central Hospital, Zibo, China; 5 Department of Cardiology, Zibo Central Hospital, Zibo, China; 6 Department of Urology, Zibo Central Hospital, Zibo, China; 7 Department of Oncology, Zibo Central Hospital, Zibo, China

**Keywords:** brain tumors, glioblastoma, epigenetics, tumor immune microenvironment, immunotherapy, T cells

## Abstract

Brain tumors such as glioblastoma remain among the most lethal and immunologically resistant cancers, in large part due to epigenetic programs that sculpt the tumor–immune microenvironment. DNA methylation, histone modifications, and chromatin remodeling do not merely drive tumor-intrinsic changes; they also profoundly reprogram immune responses, shaping antigen presentation, cytokine signaling, and immune cell recruitment. At the center of this regulation are T cells, whose effector functions are suppressed through promoter hypermethylation of antigen-processing genes, silencing of interferon pathways, and the establishment of exhaustion-specific chromatin states. Mutations such as IDH1/2 and H3K27M further reinforce these epigenetic barriers, fostering immune-cold microenvironments that disable cytotoxic T-cell activity. Emerging evidence highlights both CNS-specific adaptations, including microglial and astrocytic epigenetic programs that reinforce immune privilege, and conserved features of T cell exhaustion that mirror those in peripheral cancers. This duality underscores the need for therapeutic strategies that dismantle CNS-specific barriers while leveraging shared exhaustion programs across tumor types. Epigenetic drugs, ranging from DNA methyltransferase and EZH2 inhibitors to BET degraders and CRISPR-based epigenome editors, are beginning to restore antigenicity, reverse T cell dysfunction, and sensitize tumors to checkpoint blockade. Yet these approaches carry the paradoxical risk of disrupting CNS immune tolerance, potentially triggering harmful neuroinflammation or autoimmunity. To our knowledge, this is among the first comprehensive reviews to integrate CNS-specific immune privilege mechanisms with peripheral exhaustion pathways, providing a unified perspective on how epigenetic regulation orchestrates immune dysfunction across central and peripheral contexts. By mapping the continuum between immune evasion and global immunosuppression, we propose a conceptual framework for tailoring epigenetic-immunotherapy combinations to achieve durable antitumor immunity in the CNS.

## Graphical Abstract

Epigenetic mechanisms driving tumor immune evasion. The schematic summarizes key epigenetic alterations that contribute to immune escape in the tumor microenvironment. Central cancer cells undergo DNA hypermethylation, leading to silencing of antigen presentation and interferon signaling genes, while dysregulation of DNMTs and TET enzymes alters global methylation balance. Repressive histone modifications and chromatin remodeling further condense chromatin, restricting immune gene expression. Noncoding RNAs add another layer of repression by targeting transcripts involved in immune activation. Additionally, epigenetic reinforcement of immune checkpoint ligand expression sustains T cell exhaustion, and epigenetic reprogramming of CNS-resident or stromal cells fosters an immunosuppressive niche. Together, these interconnected mechanisms enable tumors to evade immune surveillance and resist immunotherapy.

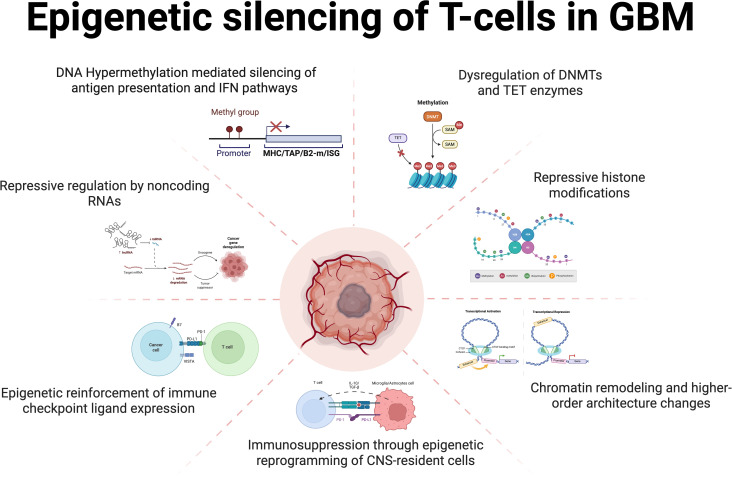

## 1 Introduction

Glioblastoma (GBM) remains one of the most lethal and treatment-resistant cancers, with a median survival of less than two years despite maximal standard therapy. Its resilience stems in large part from extraordinary epigenetic plasticity, which drives immune evasion, therapy resistance, and tumor recurrence (1–3). Epigenetic alterations, including DNA methylation, histone modifications, and chromatin remodeling, are not passive bystanders but active architects of the tumor immune microenvironment (TIME). By silencing antigen presentation, dampening interferon signaling, and reprogramming myeloid and glial cells into tolerogenic states, these programs sculpt an “immune-cold” landscape largely refractory to current immunotherapies (4–6). Recent advances in single-cell and multi-omic technologies have illuminated how these epigenetic programs vary across tumor cells, infiltrating lymphocytes, microglia, and astrocytes, revealing both parallels with peripheral cancers and adaptations unique to the immune-privileged central nervous system (CNS) (7).

Importantly, mechanistic insights are now translating into clinical strategies. Biomarker-guided interventions, such as MGMT promoter methylation predicting temozolomide (TMZ) response, already influence patient care, while next-generation approaches, including DNMT and BET inhibitors, PROTAC-based epigenetic degraders, and CRISPR-based epigenome editing, hold promise for reshaping therapy in a precise, patient-tailored manner. Yet, the therapeutic potential of epigenetic reprogramming is tempered by its risks. Epigenetic drugs act as a double-edged sword: while they can restore antitumor immunity and sensitize gliomas to checkpoint blockade, they may also destabilize CNS immune privilege and trigger harmful neuroinflammation or autoimmunity. Re-emphasizing this duality early establishes the central challenge of immuno-epigenetic therapy, balancing immune restoration with preservation of neural homeostasis.

**Figure 1:**
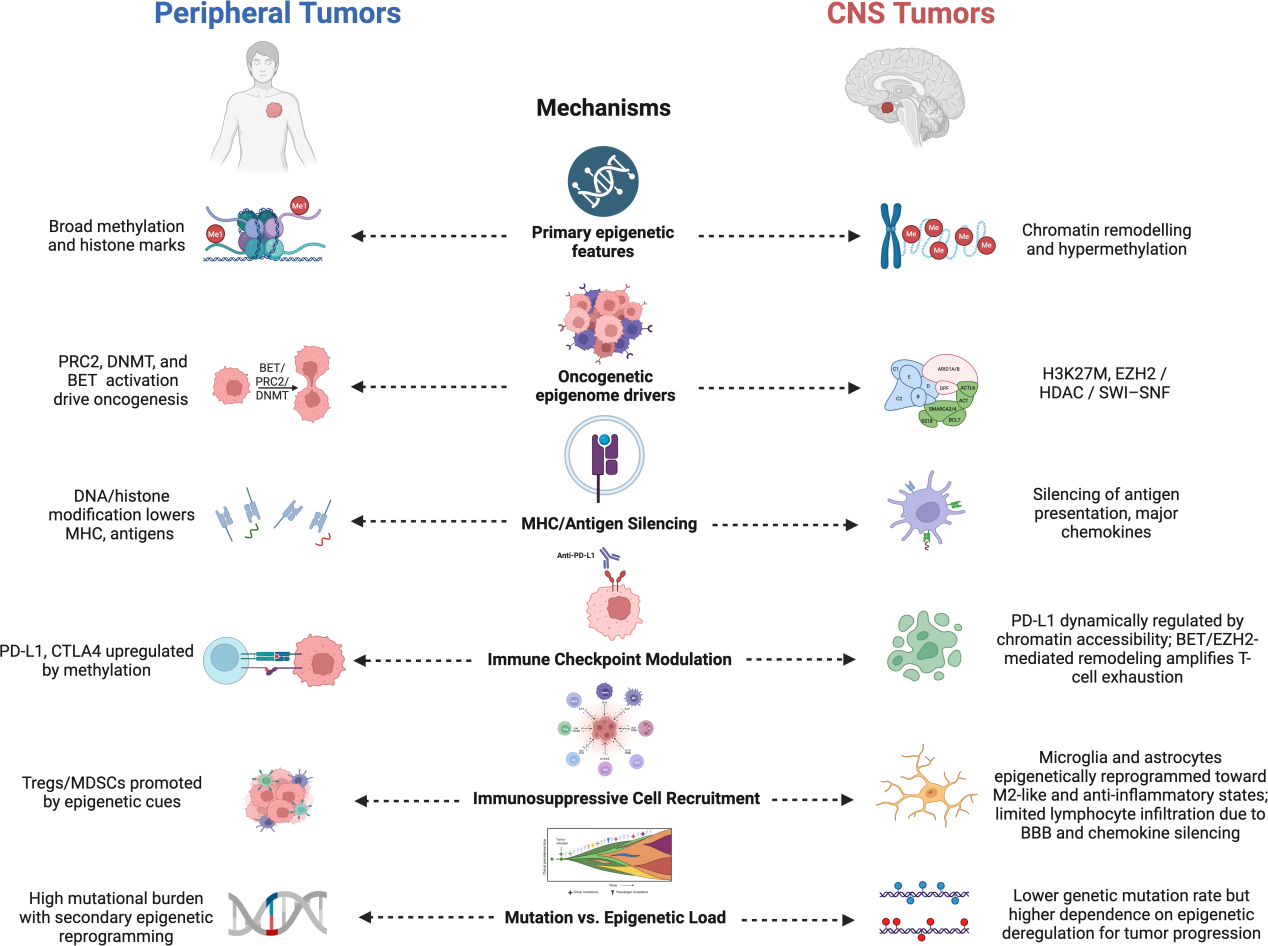
Comparative overview of epigenetic and immunological mechanisms in peripheral vs. CNS tumors. This figure contrasts how peripheral tumors and CNS malignancies, particularly glioblastoma (GBM), employ distinct epigenetic strategies for immune evasion. Peripheral tumors rely on DNA methylation and histone modifications to silence antigen-presentation and costimulatory genes, while CNS tumors integrate additional layers of suppression, including EZH2- and HDAC-mediated chromatin remodeling, SWI/SNF dysfunction, and STAT3-driven astrocytic reprogramming, that reinforce blood-brain barrier (BBB) mediated immune exclusion and glial tolerance. These mechanisms collectively underpin the enhanced immune privilege observed in CNS tumors.

To orient the reader before delving into mechanistic depth, **Table 1** summarizes the principal epigenetic alterations observed in GBM, the molecular mediators involved, and their downstream immunological consequences. This concise synthesis highlights how diverse chromatin and DNA-modifying processes converge to blunt antigen presentation, impair interferon signaling, and drive resistance to therapy. Complementing this overview, **Figure 1** contrasts immune-evasion strategies in peripheral tumors with CNS-specific adaptations. Whereas peripheral cancers primarily rely on epigenetic silencing of MHC-I/II genes and upregulation of PD-L1, brain tumors integrate additional CNS-restricted layers of control, including microglial and astrocytic chromatin remodeling, suppression of cGAS–STING signaling, and maintenance of BBB-mediated immune exclusion.

**Table 1:** Key epigenetic alterations in glioblastoma and their immunological consequences.

Epigenetic alteration	Mechanism	Immunological consequence	Reference(s)
Epigenetic remodeling by DNMT and HDAC inhibitors	Pharmacological modulation	Reactivates silenced MHC and interferon genes, enhances antigen presentation, promotes cytotoxic T-cell infiltration, and synergizes with immune checkpoint blockade; induces viral mimicry through endogenous retroelement reactivation	Topper et al. (8); Pang et al. (6); Dai et al., 2024 (9)
Non-coding RNA regulation (microRNA-148a, lncRNAs such as MALAT1, HOTAIRM1)	RNA methylation / transcriptional silencing	Modulates macrophage polarization, suppresses dendritic-cell activation, impedes T-cell recruitment, maintains glioma stem-like phenotypes, and sustains immune evasion	Ahmadov et al. (10); Guo et al., 2022 (11); Pang et al. (6)
H3K27me3 redistribution and histone methyltransferase dysregulation (EZH2, JMJD3)	Histone modification	Silences antigen-processing genes (CIITA, TAP1/2) and chemokines (CXCL9/10); promotes immune-cold TIME with reduced CD8^+^ infiltration and T-cell exhaustion	Lin et al. (12); Arenas-Ramirez et al., 2018 (13)
HMGB1 release and chromatin-associated DAMP signaling	DNA-binding protein modification	Activates innate immune receptors (TLR4, RAGE), leading to pro-inflammatory cytokine release but paradoxically fostering chronic inflammation and tumor immune tolerance	Jung et al., 2025 (14)
Histone acetylation imbalance (P300/CBP, HDAC overactivity)	Histone acetylation	Suppresses MHC and costimulatory gene expression; enhances Treg and MDSC recruitment; contributes to resistance against immunotherapy	Zhang et al. (15); Yang et al., 2023 (16)
IDH1/2 mutations and G-CIMP phenotype	Oncometabolite-driven DNA hypermethylation	Generates genome-wide CpG hypermethylation; alters myeloid cell programming and interferon signaling; associated with relative immune quiescence yet improved prognosis	de Souza et al., 2018 (17); Read et al., 2024 (18)
DNMT overexpression and global promoter methylation	DNA methylation	Silences MHC-I/II and interferon genes; downregulates antigen presentation and cytokine production; fosters durable immune escape	Lin et al. (12); Tomasi et.al; 2006 (19)
MGMT promoter methylation	DNA methylation biomarker	Inactivates DNA repair enzyme MGMT; increases sensitivity to temozolomide while modulating antigenicity; serves as predictive biomarker in GBM therapy	Mansouri et al. (20); Sahm et al., 2023 (21)

Together, these conceptual and visual frameworks set the stage for the sections that follow, which dissect how DNA methylation, histone modifications, and non-coding RNAs dynamically shape immune regulation in GBM. By bridging tumor-intrinsic and CNS-specific perspectives, this review provides a roadmap for developing precision epigenetic interventions that can overcome GBM’s formidable defenses while safeguarding the delicate immunological equilibrium of the brain. **Table 1** summarizes the key epigenetic alterations in GBM and their immunological consequences, including DNMT- and HDAC-mediated silencing of interferon genes, histone mark redistribution, and non-coding RNA–driven immune suppression, and links each to its downstream immunological consequence. Further, building on this foundation, **Figure 1** contrasts these mechanisms across contexts, highlighting how peripheral tumors and CNS tumors deploy epigenetic control differently, with the latter layering CNS-specific features such as microglial tolerance, chromatin remodeling unique to glia, and blood–brain barrier–mediated immune exclusion.

## 2 Dynamic roles of epigenetic mechanisms in brain tumors

Epigenetic mechanisms, including DNA methylation, histone modifications, and non-coding RNAs, govern gene expression without altering DNA sequence, profoundly shaping both tumor biology and immune interactions in brain cancers. These processes regulate antigen presentation, cytokine signaling, and immune cell recruitment, thereby acting as active drivers of tumor progression, immune evasion, and therapeutic response rather than passive disease markers. Together, these epigenetic layers form an interconnected regulatory framework that orchestrates immune escape, linking tumor-intrinsic chromatin dynamics to suppression of adaptive and innate immunity.

### 2.1 DNA methylation and demethylation in T cells and other immune pathways

DNA methylation is one of the most extensively studied epigenetic marks and plays a central role in both tumor biology and immune regulation in brain tumors. It typically involves the addition of a methyl group to the 5-carbon position of cytosine within CpG dinucleotides, a reaction catalyzed by DNA methyltransferases (DNMT1, DNMT3A, DNMT3B) that generally leads to transcriptional silencing of associated genes. Active DNA demethylation, in contrast, is mediated by ten-eleven translocation (TET) enzymes, which oxidize 5-methylcytosine to 5-hydroxymethylcytosine and related derivatives, enabling gene reactivation (22). These processes are highly dynamic and context-dependent, with aberrant methylation patterns contributing not only to oncogenesis but also to the modulation of anti-tumor immunity.

In brain tumors, aberrant DNA methylation patterns contribute to both oncogenesis and immune modulation (23). In glioblastoma, promoter hypermethylation of the DNA repair gene *MGMT* reduces DNA repair capacity, confers sensitivity to alkylating chemotherapy such as temozolomide, and remains one of the most robust predictive biomarkers in neuro-oncology (20). From an immunological standpoint, methylation directly regulates immune checkpoint expression: PD-L1 promoter hypomethylation has been linked to its overexpression in gliomas, enabling tumor cells to evade T cell-mediated killing (24). Similarly, hypermethylation of genes central to antigen processing and presentation, such as *TAP1* and *MHC class I*, can suppress cytotoxic T cell recognition and impair immune surveillance (25). IDH-mutant gliomas exhibit a CpG island methylator phenotype (G-CIMP), reprogramming transcriptional networks and attenuating interferon signaling, whereas H3K27M-mutant tumors show extensive chromatin alterations, reinforcing immune exclusion (26). In medulloblastomas, distinct methylation profiles define molecular subgroups, inform prognosis, and correlate with patterns of immune infiltration, underscoring their dual role as biomarkers and functional modulators of tumor immunity (27). Brain metastases further highlight the adaptive nature of methylation landscapes, as metastatic cells acquire CNS-specific methylation patterns that reduce antigenicity and promote immune exclusion (28). Beyond their biological significance, DNA methylation patterns have transformed CNS tumor diagnostics, with large-scale methylome profiling enabling high-resolution classification of brain tumors through advanced methylation classifiers, as shown by multiple reports (29–31). Such platforms can distinguish a wide range of CNS tumor categories, facilitate the recognition of novel entities, and refine or correct a substantial proportion of diagnoses, often influencing patient management (32, 33). The capacity to match diagnostically challenging tumors to reference methylation signatures using supervised online tools has made methylation profiling an integral component of contemporary neuropathology (34).

Functionally, methylation states are not static but evolve with tumor progression and treatment. Pharmacologic inhibition of DNMTs can reverse silencing at MHC and interferon-stimulated loci, partially restoring antigen presentation and enhancing responsiveness to immune checkpoint blockade, though effects remain constrained by the immune-specialized CNS microenvironment (35, 36). These tumors generally have a better prognosis but display distinct immunological features that may affect immunotherapy outcomes. Advances in machine learning applied to methylome data now allow prognostication, therapy stratification, and even real-time monitoring of treatment response (37). Together, these findings establish DNA methylation and demethylation as dynamic, multifaceted regulators of tumor–immune interactions in brain tumors, with intertwined diagnostic, prognostic, and therapeutic significance. They act not merely as passive markers of disease but as active determinants of how brain tumors develop, evade immunity, and respond to therapy.

### 2.2 Histone modifications in tumor–immune crosstalk

Histone modifications are a fundamental layer of epigenetic regulation, controlling chromatin accessibility and transcriptional activity without altering the DNA sequence. Among the numerous post-translational marks identified in brain tumors, acetylation, methylation, and phosphorylation are the most extensively studied for their impact on tumor biology, immune regulation, and therapy response. These chemical modifications fine-tune the expression of genes involved in tumor proliferation, immune surveillance, cytokine signaling, and antigen presentation.

Histone acetylation generally promotes transcription by neutralizing lysine’s positive charge, loosening chromatin structure, and facilitating transcription factor binding. This process is mediated by histone acetyltransferases (HATs) such as p300/CBP and GCN5, and reversed by histone deacetylases (HDACs) (38). In glioblastoma (GBM) and other high-grade gliomas, aberrant HDAC activity is associated with repression of MHC class I and II genes, costimulatory molecules, and interferon-responsive pathways, thereby impairing antigen presentation and facilitating immune evasion (39). HDAC overexpression also correlates with increased recruitment of regulatory T cells (Tregs) and myeloid-derived suppressor cells (MDSCs). Preclinical and early clinical studies show that HDAC inhibitors (HDACi) can reverse these effects, upregulating immune-related genes and enhancing sensitivity to immune checkpoint blockade (40).

Histone methylation has context-dependent effects determined by the specific residue and degree of methylation (mono, di, or tri). Repressive marks such as H3K27me3 and H3K9me3, catalyzed by histone methyltransferases (HMTs) like EZH2, are frequently dysregulated in gliomas and pediatric brain tumors, silencing antigen-processing genes and promoting immune exclusion (41). Loss of these marks, observed in diffuse midline gliomas and medulloblastomas, can also disrupt normal developmental programs, driving tumorigenesis. In contrast, activating marks such as H3K4me2/3, deposited by MLL family HMTs, promote transcription of immune effector genes (42). Histone demethylases (HDMs) such as KDM6B (JMJD3) can remove repressive marks, reactivating pro-inflammatory programs in microglia and infiltrating lymphocytes (43). In particular, EZH2 overexpression suppresses chemokines such as CXCL9 and CXCL10, limiting cytotoxic T cell infiltration, while pharmacologic inhibition of EZH2 restores these signals, converting immune-cold tumors into immune-inflamed states (44).

Histone phosphorylation, mediated by kinases such as MSK1/2 and Aurora kinases, influences transcription, DNA repair, and cell cycle control. In immune regulation, phosphorylation of H3S10 is linked to rapid cytokine gene activation following immune stimuli (45). In GBM, aberrant phosphorylation patterns contribute to tumor proliferation and altered inflammatory signaling. Although direct pharmacological targeting remains difficult, modulating upstream pathways such as MAPK or PI3K-AKT offers an indirect means to influence immune-relevant transcription (46).

Beyond these well-characterized marks, emerging modifications, such as lactylation, crotonylation, and succinylation, are being identified in brain tumors, though their immunological relevance is not yet fully defined (15). Overall, histone modifications operate as dynamic molecular switches that integrate oncogenic signaling with immune regulation in the CNS tumor microenvironment. Their coordinated repression of interferon signaling, antigen presentation, and chemokine networks represents a key epigenetic mechanism of immune evasion in glioblastoma.

### 2.3 Non-coding RNAs as regulators of T cell exhaustion and immune suppression in GBM

Non-coding RNAs (ncRNAs), including microRNAs (miRNAs), long non-coding RNAs (lncRNAs), and circular RNAs (circRNAs), have emerged as key epigenetic regulators in brain tumors, orchestrating transcriptional and post-transcriptional programs that shape tumor–immune interactions. Although they do not encode proteins, ncRNAs influence immune cell differentiation, cytokine and chemokine signaling, and the overall functional state of the tumor immune microenvironment (TIME) (47). In the immune-specialized setting of the central nervous system (CNS), where inflammatory responses are tightly controlled, ncRNA-mediated pathways provide tumor cells with a powerful means of modulating both local and systemic immunity.

In GBM, MicroRNAs such as miR-124 and miR-128 have been implicated in skewing tumor-associated macrophages (TAMs) toward an immunosuppressive M2 phenotype, dampening pro-inflammatory cytokine production (48). Conversely, downregulation of tumor-suppressive miRNAs such as miR-34a facilitates immune escape by upregulating PD-L1 expression on glioma cells, promoting T cell exhaustion (49). Long non-coding RNAs like HOTAIRM1 drive the expansion of myeloid-derived suppressor cells (MDSCs) in GBM via regulation of arginase-1 and inducible nitric oxide synthase (iNOS), thereby amplifying immune suppression (10). Similarly, MALAT1 upregulation promotes TGF-β secretion, fostering an immunosuppressive milieu that restricts cytotoxic T cell infiltration (50, 51). Circular RNAs are covalently closed RNA molecules noted for their stability and their role as miRNA sponges. In GBM, circ_002136 promotes VEGF-A–driven angiogenesis and indirectly suppresses dendritic cell activation by sequestering miR-138-5p, which normally targets pro-angiogenic transcripts linked to immune regulation (52). Additionally, chromatin-remodeling complexes such as SWI/SNF (ARID1A, SMARCA4) dynamically reposition nucleosomes at interferon-stimulated and antigen-presentation loci. Their dysfunction in gliomas restricts transcription factor access (IRF1, STAT1), attenuating innate immune activation and antigenicity. Collectively, ncRNAs and chromatin-remodeling pathways function as interconnected layers that fine-tune immune suppression in gliomas. Their combined dysregulation enforces a transcriptionally repressed, antigen-poor, and interferon-silent state that underlies T-cell exhaustion and immune exclusion.

**Figure 2:**
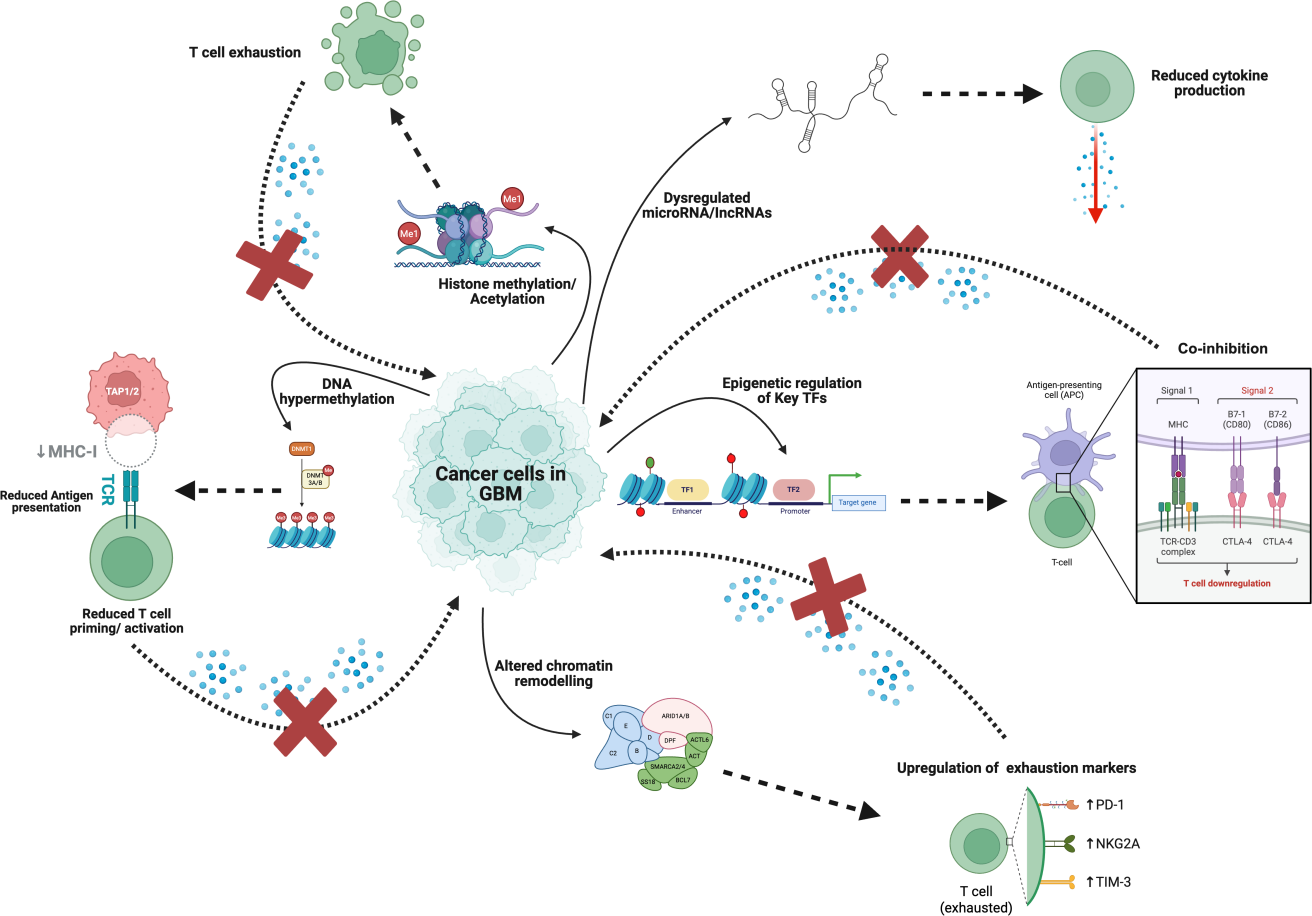
Tumor-intrinsic epigenetic mechanisms suppressing antigen presentation and T-Cell function in glioblastoma. Epigenetic dysregulation in glioblastoma (GBM) reprograms tumor cells to evade immune recognition and induce T-cell dysfunction. DNA hypermethylation and repressive histone marks (H3K27me3, H3K9me2/3) silence antigen-presentation genes (*MHC I/II*, *TAP1/2*, *CIITA*) and interferon pathway regulators, diminishing tumor visibility to cytotoxic T cells. Aberrant chromatin remodeling and dysregulated non-coding RNAs further suppress cytokine secretion and T-cell priming. Elevated PD-L1 expression and co-inhibitory signaling through PD-1, CTLA-4, and TIM-3 pathways reinforce T-cell exhaustion and immune escape. Collectively, these tumor-intrinsic epigenetic alterations establish a transcriptionally repressed, immune-cold microenvironment that undermines effective antitumor immunity.

Although these epigenetic mechanisms act through distinct biochemical pathways, they converge on a unified immunological outcome: the suppression of T-cell recognition and effector function. **Table 2** summarizes the major epigenetic regulators in GBM, including DNMTs, TET enzymes, EZH2, HDACs, and SWI/SNF complexes, and delineates how each modulates antigen presentation, interferon signaling, and T-cell effector function. Complementing these mechanistic insights, **Figure 2** provides a schematic overview of how these tumor-intrinsic epigenetic alterations, spanning DNA hypermethylation, histone modification, chromatin remodeling, and non-coding RNA regulation, collectively converge to suppress T-cell recognition and foster immune evasion in GBM.”

**Table 2:** Key epigenetic regulators in GBM, their principal molecular targets, and their immunological consequences.

Epigenetic regulator	Mechanism	Molecular targets	Effect on immune pathways	Functional outcome
DNMT1/DNMT3A/3B	DNA methylation (gene silencing)	TAP1, CIITA, MHC-I/II	Suppresses antigen presentation and limits CD8^+^ T-cell recognition	Tumor immune evasion
TET1/2/3	DNA demethylation (gene reactivation)	IFN-responsive promoters	Reactivates interferon signaling and promotes immune visibility	Enhances T-cell activation
EZH2 (PRC2 complex)	H3K27me3-mediated repression	CXCL9/10, MHC loci, STING pathway	Silences chemokine and interferon genes	Reduces T-cell infiltration
HDACs	Deacetylation of histones	MHC-II, CIITA, costimulatory molecules	Inhibits antigen presentation and cytokine expression	Supports Treg/MDSC expansion
SWI/SNF (ARID1A, SMARCA4)	Chromatin remodeling	IFN-stimulated genes, TLR signaling loci	Alters chromatin accessibility and dampens immune sensing	Restricts innate immune activation

## 3 Epigenetic regulation of the tumor immune microenvironment in brain tumors

The tumor immune microenvironment (TIME) in brain tumors is a highly specialized ecosystem shaped by continuous molecular dialogue between malignant cells, resident glia, and infiltrating immune populations. Within the CNS, this interplay is uniquely influenced by the blood–brain barrier, limited lymphatic drainage, and tightly regulated inflammatory responses. Epigenetic mechanisms serve as key transcriptional regulators within this environment, orchestrating immune cell activation states, antigen presentation, and cytokine production. Together, these processes define whether the CNS TIME becomes immunologically active or locked in a tolerogenic, “cold” state.

### 3.1 CNS-resident immune cells

Microglia are the brain’s resident immune sentinels, which are highly plastic and can polarize toward pro-inflammatory (M1-like) or anti-inflammatory (M2-like) phenotypes. In GBM, tumor-derived factors induce epigenetic remodeling of microglia, restricting antigen presentation, dampening interferon signaling, and promoting immunosuppressive gene expression. This includes HDAC-mediated deacetylation at MHC class II and pro-inflammatory loci, DNA methylation changes that repress *CIITA* (a key MHC regulator), and microRNA-driven suppression of NF-κB and STAT pathways. Cross-talk further intensifies when brain-tropic cancer cells lose the lncRNA XIST, triggering secretion of miR-503, which reprograms microglia toward an M2-like state that impedes cytotoxic T-cell recruitment (53). Notably, HDAC inhibitors can partially reverse this phenotype, shifting microglia/macrophages toward enhanced antigen presentation and pro-inflammatory cytokine release (40). While microglia are the primary innate immune effectors in the CNS, they operate alongside astrocytes, which, beyond their structural and metabolic support roles, also exert substantial influence over local immune responses. In the GBM microenvironment, reactive astrocytes undergo epigenetic reprogramming, often in parallel with microglial changes, to reinforce an immunosuppressive milieu. STAT-driven chromatin remodeling in astrocytes promotes transcription of anti-inflammatory mediators such as IL-10 and TGF-β, while silencing chemokines like CXCL9/10 that are essential for CD8^+^ T-cell recruitment (54). This shift is mediated in part by histone mark redistribution (e.g., reduced H3K27ac and increased H3K27me3) at interferon-responsive promoters. Dysregulated lncRNAs, including *GAS5*, can further modulate these transcriptional programs, sustaining conditions that support tumor persistence and limit adaptive immune activation (55). The combined epigenetic modulation of microglia and astrocytes establishes an immunosuppressive baseline within the CNS TIME, which is then reinforced by changes in infiltrating immune cells.

### 3.2 Infiltrating immune cells

Peripheral immune cells entering the CNS are met with a microenvironment already conditioned by resident glia toward tolerance. Infiltrating T cells often display a fixed “exhausted” phenotype, marked by high expression of inhibitory receptors (PD-1, TIM-3, LAG-3) and reduced effector capacity. This state is stabilized by a distinctive epigenetic signature, *de novo* DNA methylation, and repressive histone marks at effector loci (*IFNG*, *GZMB*), alongside active chromatin states at inhibitory receptor genes (56). Because these chromatin landscapes are relatively resistant to reversal by immune checkpoint blockade alone, there is growing interest in epigenetic therapies such as DNMT inhibitors, HDAC inhibitors, and BET inhibitors to reawaken T-cell cytotoxic potential and convert immune-excluded (“cold”) tumors into T-cell–inflamed (“hot”) lesions (8).

Recent multi-omic studies reveal that exhausted CD8^+^ T cells in glioblastoma share conserved chromatin architectures with those seen in chronic viral infection, including closed chromatin at effector genes (*IFNG*, *PRF1*, *GZMB*) and accessible enhancers near inhibitory-receptor loci (*PDCD1*, *LAG3*, *HAVCR2*). These exhaustion-specific “epigenetic scars,” orchestrated by transcription factors such as TOX, NR4A1, and BATF, are reinforced by DNMT3A-mediated methylation and H3K27me3 enrichment at effector promoters (57). The persistence of these chromatin programs explains why PD-1 blockade alone rarely restores durable cytotoxicity in GBM. Combinatorial approaches using DNMT or HDAC inhibitors can partially reopen effector loci, enhance interferon responsiveness, and promote a shift toward an activated T-cell phenotype.

In parallel, regulatory T cells (Tregs) within the GBM TIME maintain robust suppressive function through equally stable epigenetic programs. Accessibility at *FOXP3*, *CTLA4*, and *IL2RA* loci is reinforced by EZH2 and DNMT1-dependent chromatin modifications that preserve FOXP3 expression and transcriptional stability even under metabolic stress (58). This reinforcement enables Tregs to withstand inflammatory cues and sustain immunosuppression within the CNS. Selective inhibition of EZH2 has been shown to destabilize the FOXP3 program and potentiate antitumor immunity (59), although excessive inhibition may risk autoimmune neuroinflammation, underscoring the need for context-specific modulation rather than global blockade.

The functional recovery of T cells is further hindered by an influx of immunosuppressive myeloid cells. Monocyte-derived macrophages infiltrating GBM are frequently polarized toward an M2-like phenotype through methylation-dependent silencing of *NOS2* and *IL12B* and activating histone modifications at *ARG1* and *MRC1 (*60). Similarly, myeloid-derived suppressor cells (MDSCs) are epigenetically programmed by tumor-secreted cytokines to sustain STAT3 and C/EBPβ-driven transcriptional circuits that suppress T-cell activation. Tumor-derived non-coding RNAs, such as lncRNA RP11-361f15.2, further promote the recruitment and functional stabilization of these suppressive myeloid populations (61, 62).

**Figure 3:**
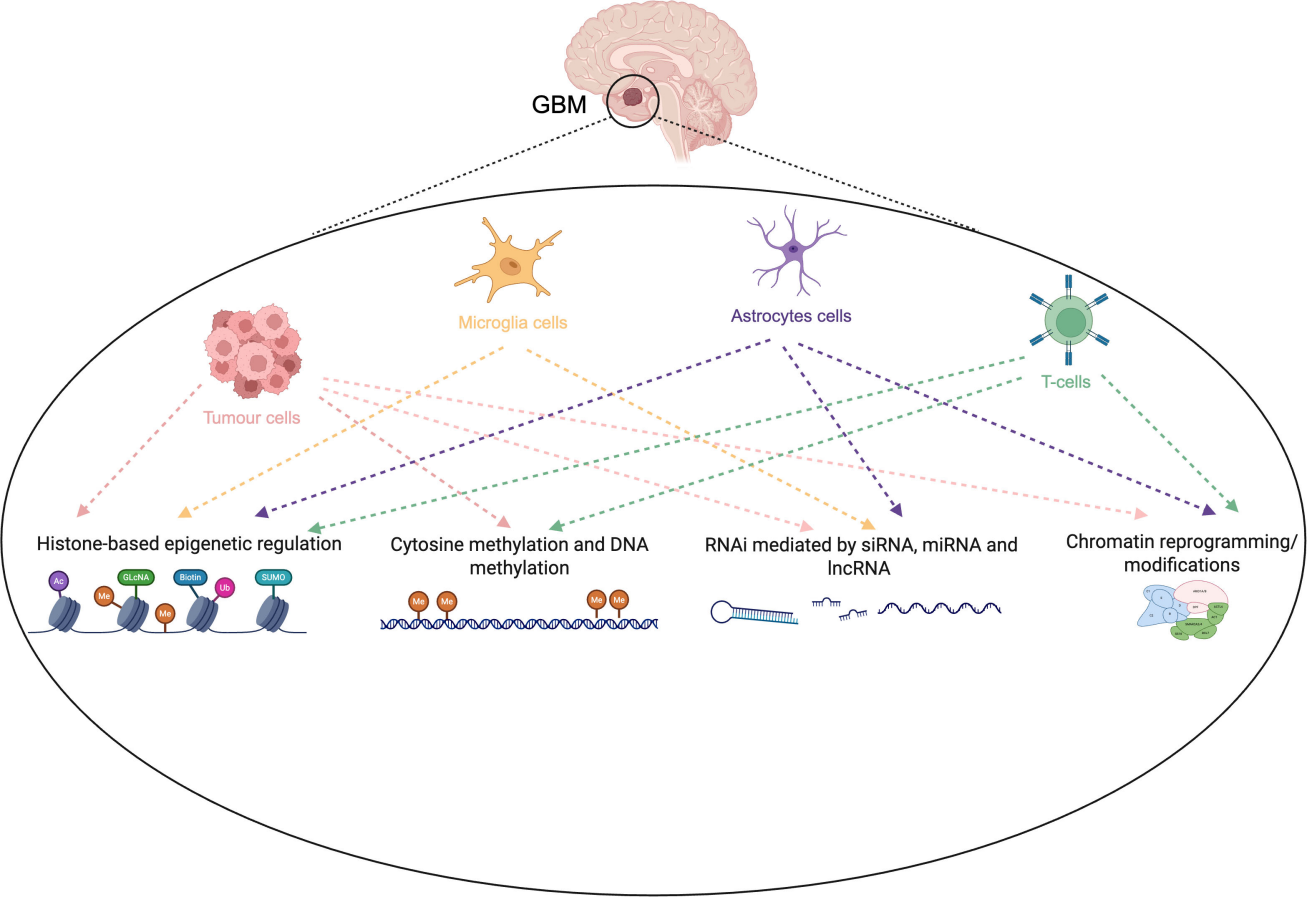
Epigenetic crosstalk between tumor, glial, and infiltrating immune cells in the glioblastoma microenvironment. Epigenetic interactions coordinate immune suppression across multiple cellular compartments in glioblastoma (GBM). Tumor cells, microglia, astrocytes, and infiltrating T cells engage in bidirectional signaling shaped by histone modifications, DNA methylation, RNA interference, and chromatin remodeling. Histone-based repression and DNA hypermethylation silence interferon and antigen-presentation pathways in tumor cells, while non-coding RNAs and chromatin reprogramming reinforce tolerogenic phenotypes in glial and immune populations. These interconnected regulatory layers collectively establish a transcriptionally repressed, immune-cold tumor microenvironment that limits T-cell activation and sustains CNS immune privilege.

Together, exhausted CD8^+^ T cells, epigenetically stabilized Tregs, and reprogrammed myeloid populations form a mutually reinforcing axis of immune suppression that amplifies and sustains the tolerogenic conditions initially established by CNS-resident glia. This cooperative epigenetic remodeling across lymphoid and myeloid compartments is a defining feature of the glioblastoma TIME and a key barrier to effective immunotherapy. **Figure 3** illustrates the intricate epigenetic crosstalk within the glioblastoma tumor immune microenvironment, mapping how histone modifications, DNA methylation, and RNA-mediated repression coordinate across CNS-resident microglia, reactive astrocytes, and infiltrating immune cells to reprogram their transcriptional states and collectively sustain an immunosuppressive TIME.

### 3.3 Cytokine and chemokine networks

The coordinated actions of CNS-resident and infiltrating immune cells in the brain tumor immune microenvironment are ultimately governed by a complex network of cytokines and chemokines whose expression is tightly regulated by epigenetic mechanisms. Tumor cells, cancer stem-like cells, and stromal populations utilize chromatin remodeling, histone modifications, and non-coding RNAs to regulate the accessibility and transcription of these key immunoregulatory genes. These changes not only influence the magnitude and type of immune infiltration but also dictate whether immune responses are stimulatory or suppressive.

In GBM, suppression of activating histone marks at *CXCL9* and *CXCL10* promoters limits CD8^+^ T-cell infiltration (15), while promoter demethylation of *CCL2* enhances recruitment of monocytes and myeloid-derived suppressor cells (MDSCs), collectively skewing the TIME toward immunosuppression (63). These changes are further reinforced by epigenetic regulators within tumor cells themselves. For example, the acetyl-lysine reader CECR2 enhances the transcription of cytokines (*CSF1*, *CSF2*, *CSF3*) *(*64) and chemokines (*CXCL1*) (65), promoting the polarization of tumor-associated macrophages (TAMs) toward an immunosuppressive phenotype. Likewise, the circadian regulator CLOCK, highly expressed in glioblastoma stem cells, induces expression of *OLFML3* and *LGMN*, which facilitate microglial infiltration and the acquisition of tumor-supportive functions (66). Building on these chromatin and transcription factor–driven mechanisms, non-coding RNAs provide an additional regulatory layer that integrates and fine-tunes cytokine and chemokine networks. MicroRNAs such as miR-21 modulate the expression of key immunoregulatory mediators, coordinating cross-talk among tumor cells, glia, and infiltrating immune populations (67). Importantly, these epigenetic programs do not act uniformly across all brain tumors; instead, they contribute to marked heterogeneity in immune landscapes.

## 4 Tailoring epigenetic therapies for glioblastoma: from biomarkers to precision editing

**Figure 4:**
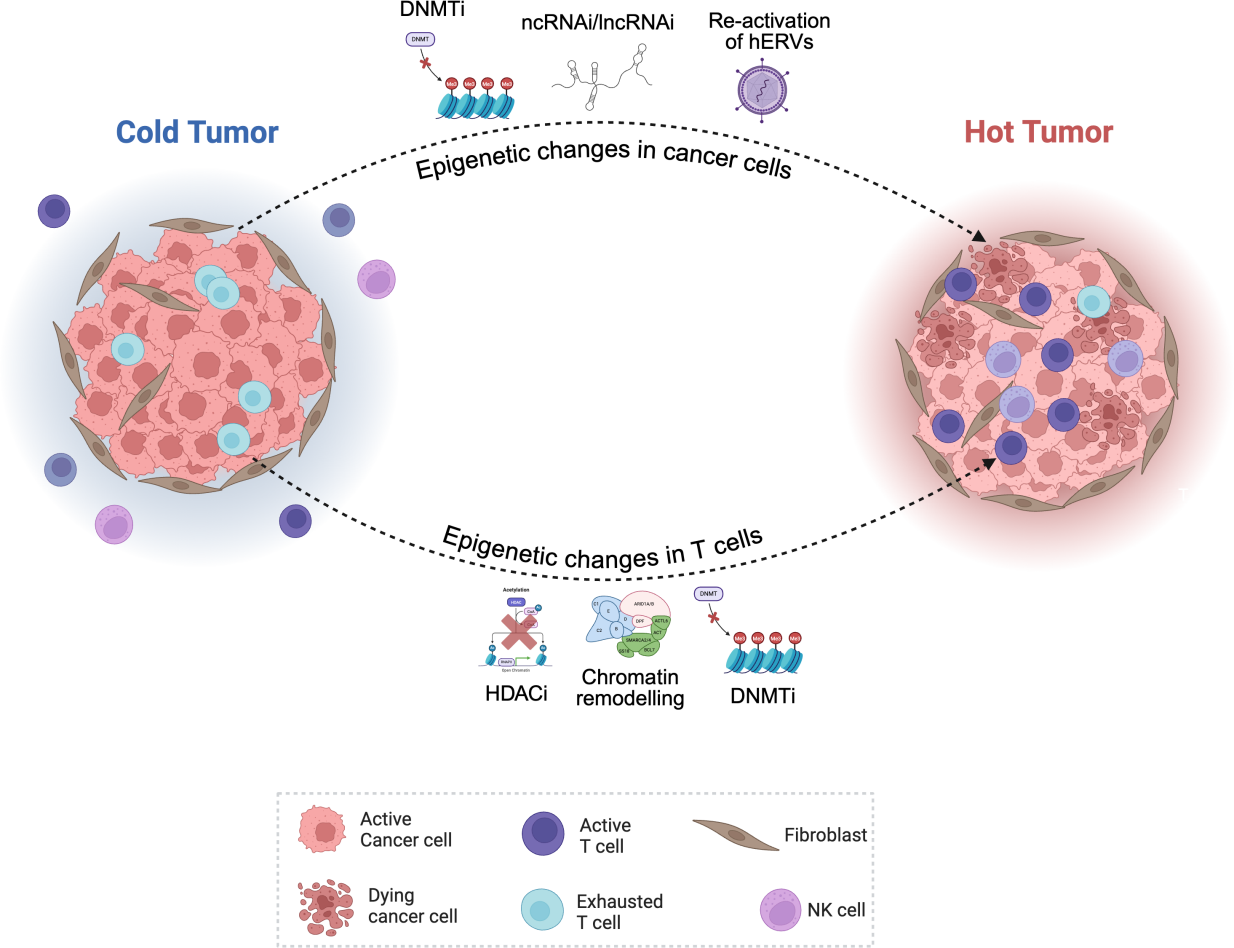
Epigenetic reprogramming strategies to convert immune-cold glioblastomas into immune-hot tumors. Epigenetic therapies reverse immune silencing in glioblastoma (GBM), transforming an immune-cold tumor into an inflamed, T-cell-active state. In untreated GBM (left), DNA hypermethylation and histone deacetylation repress *MHC I/II*, *TAP1/2*, *CIITA*, and *CXCL9/10*, blocking antigen presentation and T-cell infiltration. Inhibitors of DNMT, HDAC, and BET proteins restore chromatin accessibility and interferon signaling, re-expressing immune genes and re-establishing chemokine gradients. The resulting immune-hot tumor (right) shows enhanced CD8^+^ T-cell and NK-cell infiltration, increased antigen visibility, and improved responsiveness to checkpoint blockade.

Glioblastoma (GBM) remains one of the most formidable cancers, due largely to its exceptional epigenetic plasticity, which enables tumors to evade immune recognition, adapt to therapy, and sustain recurrence. Yet these same features create opportunities for therapeutic reprogramming. Modern immuno-epigenetic strategies aim not merely to inhibit oncogenic transcription but to reawaken silenced immune pathways, converting immune-cold GBMs into responsive, inflamed tumors. To provide a comprehensive overview of emerging interventions, **Table 3** summarizes current epigenetic therapeutics under clinical and preclinical evaluation in glioblastoma. Extending these insights, **Figure 4** visually depicts how such interventions can therapeutically reprogram an ‘immune-cold’ glioblastoma into an ‘immune-hot’ state by epigenetically restoring antigen presentation, interferon signaling, and chemokine-driven T-cell recruitment.

**Table 3:** Current landscape of epigenetic therapeutics in glioblastoma (GBM).

Drug/Class	Primary epigenetic target	Mechanistic & immunological effect	Clinical development stage	Limitations
Decitabine/5-Azacytidine (DNMTi)	DNMT1, DNMT3A/B	Reverses promoter hypermethylation; restores *MHC I/II* and antigen-processing gene expression; enhances T-cell infiltration and PD-1 blockade synergy	Phase I/II - NCT04252248, NCT04553393 (+ TMZ/anti-PD-1)	Global hypomethylation can cause systemic inflammation and myelosuppression
Guadecitabine (SGI-110)	DNMT1/3A	Next-generation DNMTi; reactivates silenced tumor-suppressor and immune genes; potentiates TMZ efficacy in recurrent GBM	Phase II - recurrent GBM studies	Short plasma half-life; limited BBB penetration
Vorinostat (SAHA)	HDAC1/2/6	Promotes histone acetylation and open chromatin; upregulates *MHC I/II* and costimulatory genes; enhances CD8^+^ T-cell activation	Phase II - NCT02137759, NCT03641053 (+ radiotherapy/pembrolizumab)	Neurotoxicity, poor CNS bioavailability
Panobinostat (LBH589)	Broad-spectrum HDACs	Induces glioma-stem differentiation, down-regulates PD-L1, augments interferon signaling	Phase I/II - NCT04308330 (+ re-irradiation/bevacizumab)	Dose-limiting fatigue, thrombocytopenia
Valproic Acid	Class I/II HDACs	Repurposed anticonvulsant; improves chromatin accessibility and radiosensitization	Phase II (+ TMZ/RT)	Modest potency; requires high CNS levels
EZH2 Inhibitors (Tazemetostat EPZ-6438, CPI-1205)	EZH2/PRC2 complex	Reverses H3K27me3-mediated repression; reactivates IFN and antigen-presentation genes; enhances T-cell recruitment	Phase I/II - NCT04179864 (IDH-wt GBM); early trials for CPI-1205 and DS-3201	Resistance via PRC2-independent pathways; hematologic toxicity
PRMT5 Inhibitors (GSK3326595)	PRMT5 (arginine methyltransferase)	Derepresses endogenous retroviruses; induces viral-mimicry and type I IFN responses; improves immune infiltration	Early-phase exploratory trials	On-target toxicity, limited CNS delivery
LSD1 Inhibitors (ORY-1001, INCB059872)	Lysine-specific demethylase 1A	Reactivates interferon and apoptotic programs; induces viral-mimicry signaling	Phase I - solid-tumor basket studies	Neurobehavioral side-effects under evaluation
BET Inhibitors (JQ1, OTX015/MK-8628)	BRD4/BET family proteins	Suppresses *MYC* and *PD-L1* transcription; enhances antigenicity and CD8^+^ chemotaxis	Phase I - NCT02296476 (recurrent GBM)	Poor BBB penetration; limited oral tolerability
ARV-825 (BRD4 PROTAC)	BRD4 degradation	Dual activity: suppresses *MGMT*, repolarizes TAMs toward M1-like state; synergizes with TMZ	Preclinical → Early clinical transition	Hepatotoxicity risk, formulation stability
IDH Inhibitors (Vorasidenib AG-881, Ivosidenib AG-120, Enasidenib AG-221)	Mutant IDH1/2 enzymes	Blocks 2-HG accumulation; reverses CpG-hypermethylation; normalizes immune signaling	Phase II - NCT03681006 (Vorasidenib + TMZ); early IDH trials	Activity confined to IDH-mutant GBM; off-target metabolic effects
PRC2/Histone Methyltransferase Modulators (Valemetostat DS-3201, Lirametostat)	H3K27 methylation pathways	Reactivates immune and differentiation programs	Early clinical	Off-target chromatin remodeling
m^6^A RNA Modulators (METTL3, FTO, ALKBH5 inhibitors)	RNA methylation machinery	Regulate PD-L1 mRNA stability and T-cell tolerance; preclinical immune-checkpoint modulation	Preclinical	Target specificity, RNA-delivery limitations
ncRNA/lncRNA Therapies (HOTAIRM1, miR-138, miR-34a)	Non-coding RNA pathways	Restore immune signaling, reduce GBM stemness, and sensitize tumors to T-cell attack	Preclinical	Delivery efficiency, transcript off-target effects
Nanocarrier and CED Platforms	Context-dependent (DNMTi, HDACi, PROTACs)	Achieve tumor-localized chromatin modulation, re-polarize TIME, enhance chemo-/immunotherapy synergy	Preclinical/proof-of-concept studies	Manufacturing scalability, spatial distribution control

### 4.1 Small-molecule epigenetic inhibitors: DNMT, HDAC, and BET pathways

Small-molecule inhibitors form the foundation of epigenetic therapy. DNA-methyltransferase inhibitors (DNMTi), such as decitabine and guadecitabine, reverse aberrant methylation and restore expression of antigen-processing genes (*TAP1*, *MHC I/II*) while weakening DNA-repair networks, thereby sensitizing GBM to alkylating chemotherapy such as temozolomide (TMZ) (68, 69). Histone-deacetylase inhibitors (HDACi) (e.g., vorinostat, panobinostat) reactivate interferon-responsive and costimulatory genes, increase T-cell infiltration, and enhance synergy with immune checkpoint blockade (40, 70).

Bromodomain and extraterminal (BET) inhibitors, such as JQ1 and OTX015, suppress acetyl-lysine “reader” proteins (e.g., BRD4) to down-regulate MGMT transcription and re-establish chemosensitivity (71). Preclinical studies demonstrate that BET inhibition can also restore chemokine signaling (e.g., CXCL9/10) and enhance T-cell recruitment (70). Despite these benefits, DNMTi and HDACi can provoke systemic inflammation and neurotoxicity, whereas BETi face limited blood–brain barrier (BBB) penetration. These representative classes, DNMTi, HDACi, and BETi, are among the most clinically advanced and are compared side by side in **Table 3**, highlighting shared immunomodulatory mechanisms and unique CNS-specific challenges, such as BBB permeability and neurotoxicity.

### 4.2 Epigenetic degraders and PROTAC-based strategies

Next-generation degraders extend beyond inhibition to targeted protein removal. Proteolysis-targeting chimera (PROTAC) BET degraders (e.g., SPP-ARV-825) achieve selective BRD4 depletion, suppress MGMT, and reprogram tumor-associated macrophages (TAMs) toward pro-inflammatory states (72, 73). Delivered through nanoparticles engineered for BBB penetration, these compounds combine chemosensitization and immune remodeling, representing a dual-action modality.

However, broad systemic activity can lead to hepatotoxicity or unintended degradation of off-target proteins. Accordingly, PROTAC-nanoparticle platforms optimized for brain delivery are being developed to localize activity and reduce collateral toxicity (72, 73).

### 4.3 CRISPR-based epigenome editing: precision reprogramming

CRISPRoff, a programmable epigenome-editing tool, introduces targeted DNA methylation and gene silencing without altering genomic sequence (74). In GBM, CRISPRoff-mediated MGMT silencing produced up to a 100-fold increase in TMZ sensitivity in preclinical models, with effects lasting across multiple cell divisions (12). Beyond MGMT, multiplexed CRISPRoff designs can target BRIP1 and FANCE, broadening sensitivity to alkylating agents such as lomustine (CCNU) (12).

Emerging RNA-guided methylation/demethylation systems further expand this paradigm, using engineered RNA-protein complexes to direct histone or DNA modifications at specific loci. Combined with single-cell and multi-omic tumor profiling, these tools enable patient-specific epigenetic programming.

Delivery remains a challenge in the CNS: nanoparticle, viral (AAV), and lipid-based platforms have achieved BBB penetration and localized silencing of target genes (12, 73). Still, off-target methylation and immune activation remain potential risks, necessitating transient expression systems and local delivery routes to maintain CNS safety.

### 4.4 Combination therapies: epigenetic modulation and checkpoint blockade

Even after overcoming chemoresistance, GBM frequently remains refractory due to a profoundly immunosuppressive TME. Epigenetic agents can “heat up” these immune-cold tumors by reactivating antigen presentation, interferon signaling, and chemokine gradients that drive T-cell recruitment.

DNMTi re-express endogenous retroviruses and cancer-testis antigens, triggering type I interferon “viral-mimicry” responses. EZH2 and HDAC inhibitors restore MHC I/II pathways and costimulatory signals, increasing CD8^+^ and CD4^+^ T-cell recognition (70). In GBM models, EZH2 blockade enhanced antigen presentation and T-cell trafficking, synergizing with PD-1 inhibition to extend survival (70). Similarly, DNMTi + ICI combinations improved infiltration and cytotoxicity in previously checkpoint-refractory tumors.

Beyond T cells, BET degraders and HDACi re-educate TAMs toward pro-inflammatory phenotypes, increase NK-cell activation, and restore cGAS–STING signaling (73, 75). Nevertheless, overstimulation can risk neuroinflammation, underscoring the need for careful dose-scheduling and CNS-focused delivery.

### 4.5 Nanotechnology and advanced delivery systems: overcoming the blood–brain barrier

The BBB severely limits many epigenetic agents. Nanoparticle carriers, including liposomes, polymeric nanoparticles, and lipid–polymer hybrids, are designed to encapsulate DNMTi, HDACi, or BET degraders, protecting them from degradation and enabling receptor-mediated CNS entry (e.g., via transferrin receptors) (73). SPP-ARV-825-loaded nanoparticles exemplify this approach, suppressing MGMT, inhibiting proliferation, and repolarizing TAMs (73).

Viral (AAV) and non-viral lipid vectors now deliver CRISPRoff constructs for localized, locus-specific editing (12). Convection-enhanced delivery (CED) bypasses the BBB entirely, infusing epigenetic drugs or vectors directly into tumors under stereotactic control, achieving high intratumoral concentrations while limiting peripheral toxicity (12, 73). Together, these platforms address both efficacy and safety: targeted CNS delivery enhances potency, reduces systemic inflammation, and enables combinatorial use with chemotherapy, radiation, and immunotherapy. Innovative delivery systems described here parallel the advances summarized in **Table 3**, where nanocarrier and CED-based approaches address the intrinsic limitations of systemic epigenetic therapy.

### 4.6 Multi-omics and AI-guided personalization of epigenetic therapy

GBM’s extreme heterogeneity necessitates individualized intervention. Integrating multi-omic profiling (genomic, epigenomic, transcriptomic, proteomic, and metabolomic) with AI-driven analytics can match patients to optimal epigenetic modalities. Single-cell scATAC-seq and scRNA-seq studies reveal glioma stem-like populations enriched in EZH2 and BRD4, alongside exhausted T-cell clusters with stable chromatin scars (76).

Machine-learning models now combine MGMT promoter methylation features with immune and transcriptomic data to refine predictions of TMZ response (77). This approach underlies precision trials such as ATTRACT (NCT06512311), which uses patient-derived tumor cultures for ex vivo screening of epigenetic agents to personalize therapy in MGMT-unmethylated GBM (78).

Ultimately, integrating multi-omics, AI, and functional screening allows epigenetic therapies to be deployed rationally rather than empirically, improving efficacy while minimizing toxicity (76–78).

Overall, these advances highlight how epigenetic therapy for glioblastoma is moving beyond broad, empiric interventions toward precisely tailored, mechanistically guided strategies. From dismantling chemotherapy resistance and introducing locus-specific epigenome editing tools, to harnessing synergistic combinations with immunotherapy and optimizing delivery across the blood–brain barrier, each approach reflects a shift toward personalization. The integration of multi-omics and AI-guided analytics further ensures that therapy design can account for intratumoral heterogeneity and patient-specific vulnerabilities. Crucially, these translational efforts not only aim to enhance therapeutic efficacy and overcome resistance but also to minimize toxicity and preserve CNS integrity, addressing the delicate balance between restoring antitumor immunity and avoiding harmful neuroinflammation. As these strategies progress into clinical trials, they underscore a broader paradigm shift: epigenetic interventions in brain tumors are no longer ancillary tools but are emerging as central, precision-directed components of next-generation therapy.

## 5 Challenges and divergent views in immuno-epigenetic reprogramming of brain tumors

Although considerable progress has been made in understanding how epigenetic mechanisms regulate immune responses in brain tumors, several fundamental challenges and divergent viewpoints remain to be solved. These debates not only reflect the inherent complexity of the tumor immune microenvironment (TIME) but also illustrate the difficulties of translating immuno-epigenetic findings into effective therapies.

### 5.1 Antigen-specific evasion versus global immunosuppression: What matters most for T cells?

A central question in the immuno-epigenetics of brain tumors is whether epigenetic dysregulation primarily drives selective antigenic silencing (79), or enforces global immune paralysis (4). Current evidence suggests that these mechanisms lie on a continuum rather than being mutually exclusive, with tumors flexibly deploying both targeted and widespread epigenetic strategies to subvert immune surveillance, depending on the tumor genotype, stage, and microenvironmental context.

Selective antigenic silencing is most evident in glioblastoma (GBM) hypermethylation or Polycomb occupancy at *CIITA*, *NLRC5*, *TAP1/2*, and *HLA-A/B* loci downregulates MHC-I/II expression without altering other immune programs (79, 80). These tumors retain residual immune responsiveness yet evade direct cytotoxic recognition, a state sometimes termed “cold but not desert.” Pharmacologic inhibition of DNMTs or EZH2 can reverse this localized repression, reactivating antigen presentation and restoring checkpoint responsiveness (81).

In contrast, global immunosuppression extends beyond discrete loci to a broad reconfiguration of the TIME. IDH1/2-mutant gliomas and H3K27M midline tumors exemplify this mode, where metabolites such as 2-hydroxyglutarate or global H3K27me3 redistribution repress interferon pathways, chemokines (CXCL9/10), and STING signaling (82–84). These widespread effects dampen both innate and adaptive immunity, producing an immunologically “desert” microenvironment refractory to immunotherapy.

Emerging data suggest these processes may coexist dynamically. GBM cells initially exploit antigenic silencing, but under therapeutic or metabolic pressure, transition to global suppression states. Clinically, this has implications for therapy selection: tumors dominated by targeted evasion may respond best to antigenicity-restoring DNMT/EZH2 blockade, whereas globally suppressed tumors may require combination strategies including STING agonists or myeloid-directed therapies (84, 85).

### 5.2 Divergent views: CNS-specific programs or peripheral parallels in T cell exhaustion?

Another ongoing debate concerns whether immune dysregulation in GBM reflects CNS-specific epigenetic adaptation or shared exhaustion programs observed in peripheral cancers. While core regulators such as DNMT3A, TOX, and NR4A1 define exhausted T-cell chromatin across tissues, brain tumors embed these mechanisms within the unique context of CNS immune privilege. Microglia, for instance, adopt stable M2-like chromatin states maintained by EZH2-mediated H3K27me3, while astrocytes silence interferon promoters via STAT3-drive**n** histone remodeling, restricting T-cell recruitment (86). Meanwhile, infiltrating CD8^+^ T cells in GBM exhibit exhaustion signatures (closed *IFNG/GZMB*, open *PDCD1/LAG3*) similar to those in chronic viral infection, but with additional repression of migration and metabolic genes, a CNS-specific adaptation limiting their persistence (87).Thus, the “divergent view” is narrowing toward a hybrid model: shared exhaustion architecture layered atop CNS-specific suppression. This perspective explains the partial responsiveness of GBM to therapies successful in peripheral cancers and highlights the need for interventions that simultaneously reverse T-cell exhaustion and dismantle glial tolerance circuits (86, 87).

### 5.3 Dual-edged epigenetic drugs: Restoring immunity or risking CNS autoimmunity?

Epigenetic therapies in brain tumors exemplify a double-edged sword: reactivation of immune pathways can restore antitumor surveillance but also risk destabilizing CNS immune tolerance. DNMT and HDAC inhibitors reopen silenced MHC and interferon genes (36), and EZH2/BET blockade amplifies chemokine production and T-cell infiltration (81, 85), yet excessive activation may provoke autoimmune neuroinflammation.

Mechanistic studies in animal models show that loss of EZH2 in Tregs impairs their suppressive stability, while DNMT inhibition can induce autoreactive Th2 cell expansion (88, 89). Clinical parallels arise from systemic demethylating agents that occasionally trigger lupus-like or CNS inflammatory syndromes. This underscores the importance of spatially restricted drug delivery, biomarker-guided dosing, and integration of nanocarriers or convection-enhanced delivery to confine immune reactivation within the tumor compartment.

Moving forward, defining quantitative thresholds of immune reawakening versus autoimmunity will be critical. Real-time monitoring of epigenetic and cytokine biomarkers may help maintain this equilibrium, achieving robust antitumor immunity while preserving the delicate architecture of neural tolerance.

### 5.4 Conceptual synthesis: the continuum of epigenetic immune regulation

**Figure 5:**
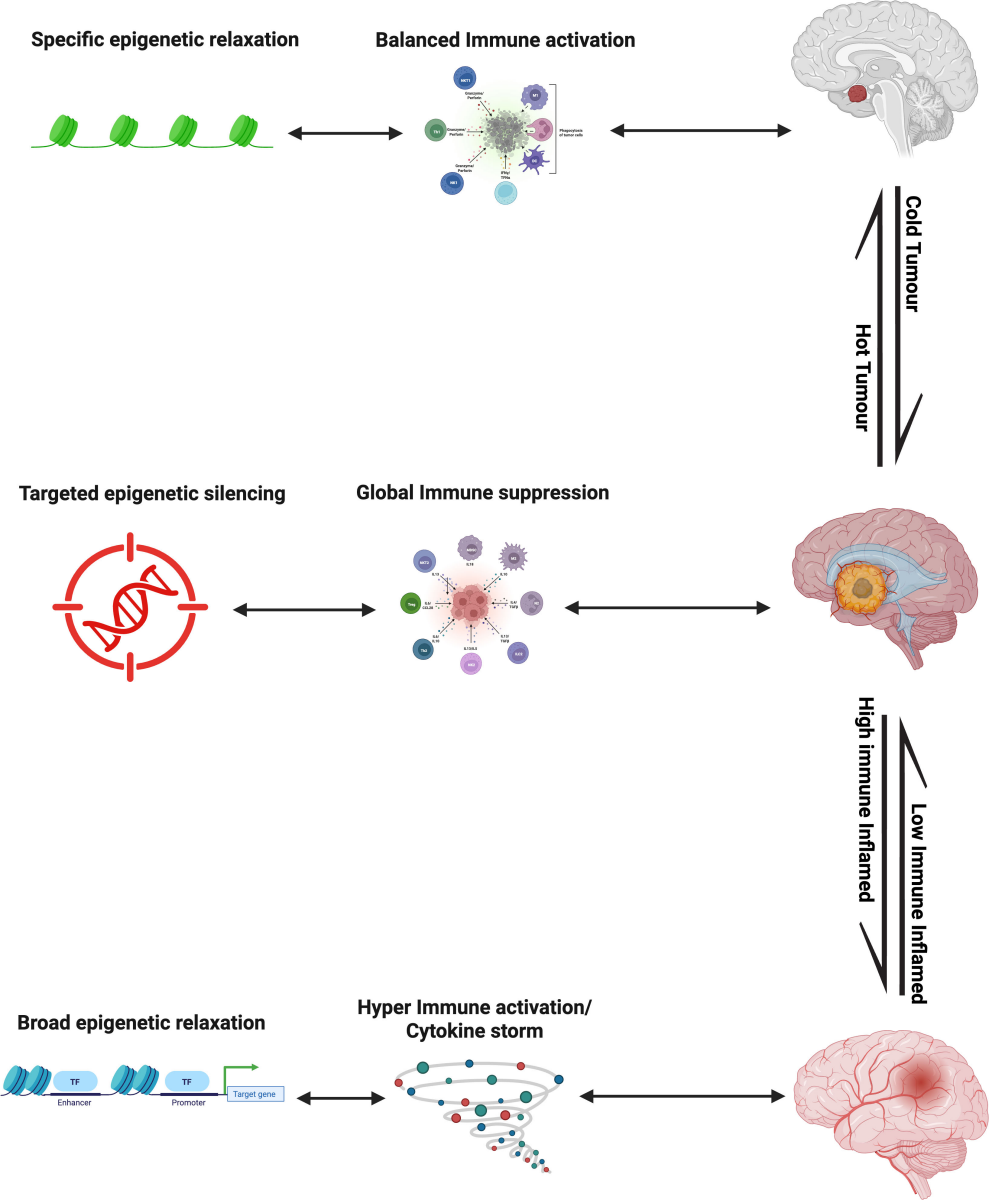
The continuum of epigenetic immune regulation in glioblastoma. The immuno-epigenetic state of glioblastoma evolves along a continuum from immune evasion to neuroinflammation. At one end, targeted epigenetic silencing through promoter hypermethylation and repressive histone marks selectively suppresses antigen-presentation and interferon genes, leading to localized immune escape. As repression expands, global immune suppression emerges, characterized by widespread chromatin condensation, cytokine silencing, and exclusion of immune cells from the tumor microenvironment. Therapeutic intervention with DNMT and HDAC inhibitors induces controlled immune reactivation, reopening immune loci and restoring antigenicity. However, excessive or unregulated chromatin relaxation can provoke hyperactivation and cytokine storm, disrupting CNS immune tolerance and causing neuroinflammation. This conceptual continuum underscores the importance of maintaining an optimal “immune window” that maximizes antitumor immunity while preserving neural integrity.

The dynamic interplay between tumor evolution and therapeutic intervention in glioblastoma (GBM) can be conceptualized as a continuum of epigenetic immune regulation (**Figure 5**). At one end is targeted silencing, where promoter hypermethylation and repressive histone marks selectively suppress antigen-presentation and interferon pathway genes, enabling immune evasion without disrupting global immune competence. With disease progression, these localized changes expand into global immunosuppression, characterized by widespread chromatin remodeling that shuts down interferon signaling, chemokine expression, and T-cell recruitment. This shift establishes an immune-desert microenvironment reinforced by metabolic and epigenetic feedback loops. Therapeutic reactivation represents the reversal of these repressive programs. DNMT, HDAC, and EZH2 inhibitors can reopen immune gene loci, restore antigen presentation, and reinitiate T-cell infiltration, transforming an immune-cold tumor into a partially inflamed state. However, excessive or uncontrolled epigenetic reprogramming risks overactivation and autoimmunity, where heightened interferon signaling or loss of regulatory T-cell restraint disrupts CNS immune tolerance and provokes neuroinflammation. This conceptual progression is illustrated in **Figure 5**, which depicts the continuum of epigenetic immune regulation in glioblastoma, from targeted gene silencing and global immunosuppression to therapeutic reactivation and, ultimately, hyperimmune activation. Complementing this model, **Table 4** organizes the key epigenetic mechanisms, corresponding immune states, measurable biomarkers, and clinical risks that define each stage of the continuum, providing a practical framework for identifying the optimal therapeutic ‘immune window’.

**Table 4:** Opportunities and risks in emerging immuno-epigenetic therapeutic strategies for glioblastoma (GBM).

Therapeutic/Functional aspect	Key opportunities	Major risks/limitations
DNMT/HDAC Inhibitors	Restore antigen presentation, enhance interferon signaling, sensitize tumors to ICIs	Global immune activation, cytokine overproduction, risk of CNS inflammation
EZH2/PRMT5 Inhibitors	Reactivate silenced IFN/chemokine pathways and reverse myeloid immunosuppression	Myelosuppression, off-target epigenetic reprogramming, and resistance via PRC2-independent pathways
BET Inhibitors/PROTACs	Suppress *MGMT*-driven resistance, downregulate *PD-L1*, and enhance T-cell chemotaxis	BBB penetration limitations, hepatotoxicity, and transcriptional compensation mechanisms
CRISPRoff/RNA-Guided Editors	Locus-specific gene silencing or activation with reversible control	Off-target methylation or demethylation; potential autoimmunity if tolerance genes disrupted
Nanotechnology/CED Delivery Platforms	Achieve confined CNS drug delivery, minimize systemic toxicity, and improve local drug concentration	Heterogeneous intratumoral distribution, invasive procedures, scalability of formulation
AI-Driven and Multi-Omic Personalization	Enable biomarker-guided, adaptive treatment design and dynamic response monitoring	Data variability, lack of standardization, and ethical constraints in predictive modeling
Immunogenicity	DNMTi and HDACi upregulate *MHC* and tumor antigens, improving immune visibility	Heterogeneous clonal response; risk of immunoediting and tumor immune escape
Combination Therapy	Synergy with ICIs, CAR-T cells, and oncolytic viruses enhances antitumor immunity	Cytokine release syndrome, neurotoxicity, and cumulative inflammatory burden
TME Remodeling	Suppression of PD-L1, IDO1, and M2-polarization enhances anti-tumor immunity	Off-target immune activation, potential CNS autoimmunity
Drug Delivery and BBB Penetration	Certain epigenetic drugs (e.g., guadecitabine) and nanocarriers cross the BBB efficiently	Suboptimal intratumoral concentration; rapid clearance or sequestration in non-target tissue
Biomarkers and Patient Stratification	Genes like *ZBTB6* and *DAXX* correlate with epigenetic therapy response; MGMT methylation guides treatment selection	Lack of validated biomarkers; intra-tumoral heterogeneity undermines predictive accuracy
Therapeutic Durability	Combination regimens yield prolonged survival and immune memory in preclinical models	Transient benefit with antigen escape or epigenetic re-silencing; need for maintenance dosing

This continuum underscores the importance of precision in immuno-epigenetic therapy, reawakening immune surveillance without compromising neural homeostasis. Defining and maintaining this optimal “immune window” is crucial for achieving durable tumor control while preserving CNS integrity.

## 6 Discussion

The study of immuno-epigenetic regulation in glioblastoma (GBM) is rapidly transitioning from descriptive biology to translational innovation. The emerging evidence underscores that epigenetic plasticity within the CNS functions as both a barrier and a therapeutic opportunity, allowing gliomas to suppress immune activation yet providing a lever to reawaken antitumor immunity. This duality defines the “double-edged sword” of epigenetic therapy: the same mechanisms that restore immune visibility and sensitize tumors to checkpoint blockade can, if overactivated, disrupt CNS immune privilege and provoke neuroinflammation.

The distinction between CNS-specific immune privilege and shared exhaustion mechanisms across peripheral cancers continues to refine therapeutic strategy. While T-cell exhaustion signatures, marked by stable methylation at *IFNG* and *GZMB* and accessible enhancers at *PDCD1* and *LAG3*, mirror peripheral tumors, GBM uniquely integrates these programs within glial-driven tolerance networks. Microglial and astrocytic epigenetic remodeling suppresses interferon and chemokine signaling, creating a layered defense that renders GBM resistant to single-agent immunotherapy. Future success will depend on addressing both arms of this suppression, reactivating exhausted lymphocytes while disarming glial tolerance, through precisely timed and spatially restricted interventions.

**Figure 6:**
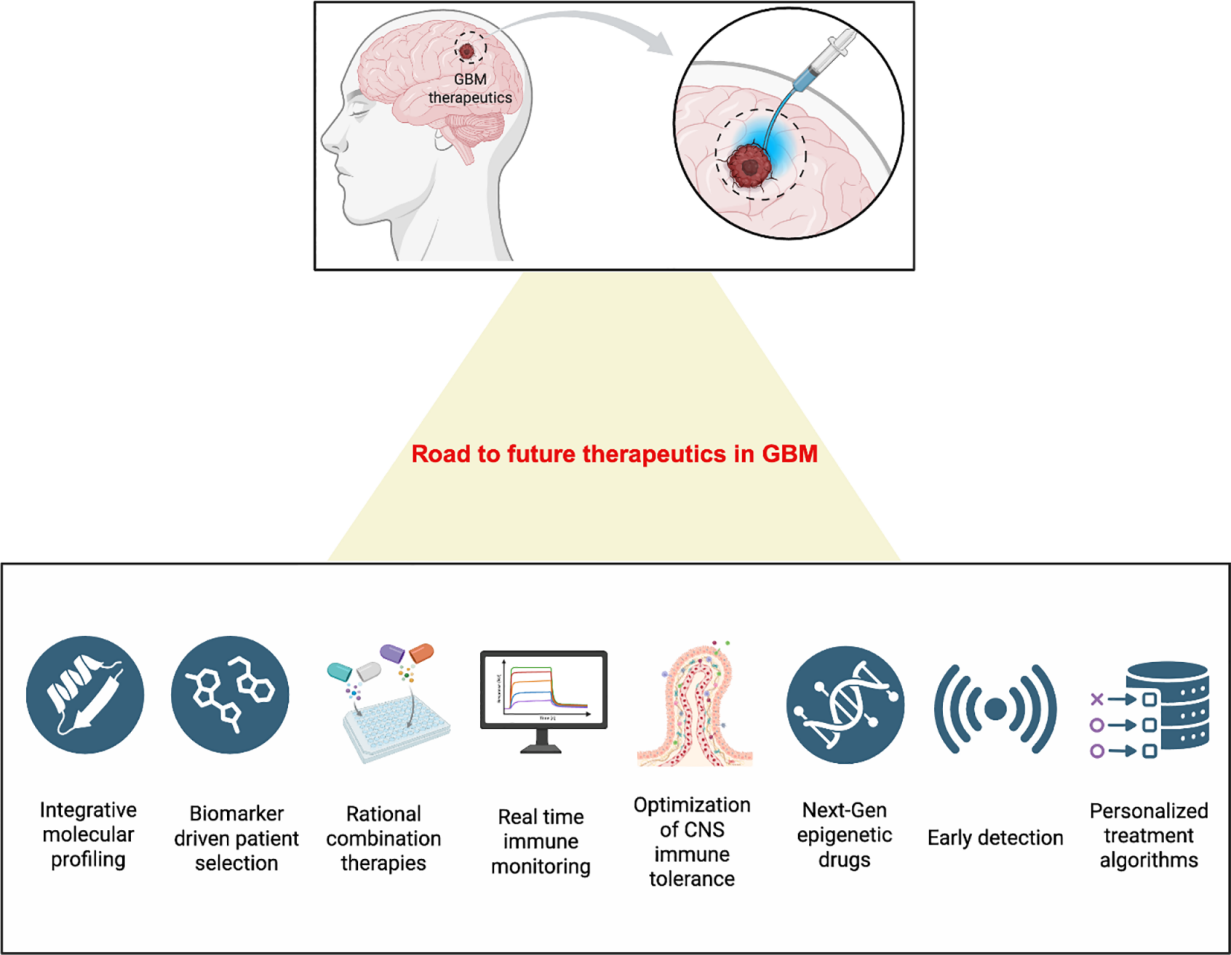
Future roadmap for precision immuno-epigenetic therapy in glioblastoma. The next generation of glioblastoma (GBM) therapeutics will integrate molecular precision with immune modulation. A unified roadmap envisions the combination of integrative molecular profiling and biomarker-driven patient selection to guide rational epigenetic–immunotherapy combinations. Real-time immune monitoring and optimization of CNS immune tolerance will ensure controlled immune activation without neurotoxicity. Development of next-generation epigenetic drugs, early detection systems, and AI-guided personalized treatment algorithms will enable dynamic, adaptive, and patient-specific management of GBM. This framework represents a shift from static, empirical therapy toward precision-guided immuno-epigenetic reprogramming for durable and CNS-safe tumor control.

The accelerating convergence of multi-omics, spatial transcriptomics, and AI-based predictive modeling is redefining how these interventions can be personalized. Spatially resolved single-cell epigenomic maps now reveal not only which immune pathways are silenced, but also where within the tumor they are silenced. When integrated with computational modeling, these maps can guide biomarker-driven drug selection, linking molecular subtype, immune phenotype, and therapeutic vulnerability. Such datasets form the foundation of patient-specific immuno-epigenetic atlases, a necessary step toward individualized therapy planning. **Figure 6** presents a forward-looking roadmap for the development of immuno-epigenetic therapies in GBM.

Technological advances are similarly expanding the therapeutic toolkit. CRISPRoff-based editors enable locus-specific methylation control, re-silencing repair genes like *MGMT* or reactivating interferon loci without global chromatin disruption. Nanoparticle and convection-enhanced delivery (CED) systems can localize these interventions within the tumor parenchyma, minimizing systemic exposure. Together, these innovations are transforming the conceptual landscape, from broad, systemic interventions to precision-directed, CNS-safe immuno-epigenetic modulation.

Despite this optimism, the field must navigate biological and ethical challenges. Epigenetic reactivation thresholds, how much immune stimulation is sufficient yet safe, remain undefined. Overactivation could erode CNS immune privilege, induce neurotoxicity, or disrupt neuronal–glial homeostasis. Conversely, underactivation risks incomplete immune awakening and therapeutic relapse. Real-time monitoring of chromatin accessibility, cytokine signaling, and cerebrospinal biomarkers could allow adaptive control of treatment intensity, guided by AI-driven feedback systems. The biomarker-guided and multi-omic feedback principles proposed here align with the opportunities and limitations categorized in **Table 4**, underscoring the translational importance of integrating immune monitoring with epigenetic modulation.

Ultimately, the future of GBM therapy lies in achieving precision without collateral activation. Rather than treating epigenetic therapies as immune accelerants, they must be deployed as calibrators, rebalancing immune and epigenetic networks to achieve sustained tumor control while preserving neural integrity. This paradigm marks a conceptual closure to the central theme, the promise and peril of manipulating the epigenome in the most immune-sensitive organ of the human body.
